# Antimicrobial Activity of *Origanum vulgare* Essential Oil against *Staphylococcus aureus* and *Escherichia coli*

**DOI:** 10.3390/ph17111430

**Published:** 2024-10-25

**Authors:** Sonia Tejada-Muñoz, Denny Cortez, Jesús Rascón, Segundo G. Chavez, Aline C. Caetano, Rosa J. Díaz-Manchay, Julio Sandoval-Bances, Sonia Huyhua-Gutierrez, Lizandro Gonzales, Stella M. Chenet, Rafael Tapia-Limonchi

**Affiliations:** 1 Instituto de Investigación de Enfermedades Tropicales, Universidad Nacional Toribio Rodríguez de Mendoza de Amazonas, Chachapoyas 01001, Peru; dennylizbethc@gmail.com (D.C.); jesus.rascon@untrm.edu.pe (J.R.); julio.sandoval@untrm.edu.pe (J.S.-B.); sonia.huyhua@untrm.edu.pe (S.H.-G.); stella.chenet@untrm.edu.pe (S.M.C.); 2 Instituto de Salud Integral Intercultural, Facultad de Ciencias de la Salud, Universidad Nacional Toribio Rodríguez de Mendoza de Amazonas, Chachapoyas 01001, Peru; 3 Instituto de Investigación para el Desarrollo Sustentable de Ceja de Selva (INDES-CES), Universidad Nacional Toribio Rodríguez de Mendoza de Amazonas, Chachapoyas 01001, Peru; segundo.quintana@untrm.edu.pe (S.G.C.); alinE.caetano@untrm.edu.pe (A.C.C.); 4 Departamento de Ciencias de la Salud, Escuela de Enfermería, Universidad Católica Santo Toribio de Mogrovejo, Chiclayo 14012, Peru; rdiaz@usat.edu.pe; 5 Dirección Regional de Salud de Amazonas, Laboratorio de Referencia Regional, Chachapoyas 01001, Peru; lgonzalesc@hotmail.com; 6 Facultad de Medicina, Universidad Nacional Toribio Rodríguez de Mendoza de Amazonas, Chachapoyas 01001, Peru

**Keywords:** antimicrobial activity, essential oil, *Origanum vulgare*, *Staphylococcus aureus*, *Escherichia coli*, Amazonas

## Abstract

**Background/Objectives**: *Oreganum vulgare* essential oil (OEO) is safe, effective, multifunctional, and widely used. This study aimed to evaluate OEO’s chemical composition and antimicrobial activity in vitro against *S. aureus* and *E. coli*. **Methods**: The composition of OEO was determined by gas chromatography–mass spectrometry (GC-MS). **Results**: Compounds included monoterpenes with known antimicrobial activity, such as 2-menthen-1-ol (36.33%), linalyl acetate (9.26%), terpinene-4-ol (9.01%), 4-thujanol (6.33%), menthen (5.81%), sabinene (5.18%), and carvacrol methyl ether (5.14%). **Conclusions**: OEO had a strong antimicrobial activity with a minimum inhibitory concentration (MIC) of 1.90 mg/mL for *S. aureus* and 0.49 mg/mL for *E. coli* after 18 h incubation. The minimum bactericidal concentration (MBC) was 7.9 mg/mL against *S. aureus* and 0.99 mg/mL against *E. coli*. Thus, OEO could be used as a natural antimicrobial against *S. aureus* and *E. coli* infections.

## 1. Introduction

Essential oils are potential natural remedies against pathogenic bacteria like *Escherichia coli* and *Staphylococcus aureus*. *E. coli*, a common Gram-negative bacterium, has developed resistance to a wide range of antibiotics, including critically important ones like ciprofloxacin and even carbapenems, often used as last-resort treatments. This resistance is largely due to the bacteria’s ability to acquire and spread resistance genes through plasmids and other mobile genetic elements [1]. On the other hand, *S. aureus*, particularly the methicillin-resistant strain (MRSA), has become notorious for its resistance to methicillin and other beta-lactam antibiotics. MRSA’s resistance is primarily attributed to acquiring the mecA gene, which encodes a modified penicillin-binding protein that reduces the effectiveness of beta-lactam antibiotics [2].

*S. aureus* can cause various diseases, from skin infections to pneumonia and sepsis; however, antibiotic resistance could complicate its treatment. Infections caused by antibiotic-resistant strains often occur in epidemic waves due to MRSA [1]. Meanwhile, although *E. coli* is part of the normal flora, some strains, such as *E. coli* O157:H7, cause diarrhea, stomach pain, nausea, and vomiting in healthy and immunocompromised individuals [2]. In recent decades, the inappropriate use of antibiotics has increased antimicrobial resistance (AMR) in both commensal and pathogenic bacteria. Overuse and misuse of antibiotics, including inappropriate prescriptions and incomplete courses of treatment, accelerate this process by creating selective pressure that favors resistant strains. The emergence of resistant bacteria poses significant challenges for public health, leading to prolonged illness, increased healthcare costs, and higher mortality rates. This issue has been observed across various production fields. It has been reported that foodborne bacteria found during food manufacturing also contribute to this problem, posing a threat to public health [3,4]. The key factors that contribute to the virulence and pathogenicity of these bacteria in comparison to others include their ability to form biofilms, activate exotoxins, and express specific genes such as the two-component arlRS system, α-hemolysin hla, nuclease (nuc1 and nuc2), and psmα (phenol-soluble α-modulins). These factors make them resistant to the latest generation of antibiotics [5,6,7].

Essential plant oils (EPOs) have been used to combat the harmful effects of bacteria. EPOs have various beneficial properties, including high antioxidant and antimicrobial effects [8]. Plus, they are promising for developing safe antimicrobial agents or compounds [9]. Many plants have been researched for their antibacterial, antifungal, antiviral, or anti-inflammatory properties. Some antioxidant extracts and essential oils [10] are safe, effective, and versatile alternative treatments [11]. The use of aromatic plants in traditional medicine is gaining renewed recognition for their ability to alleviate pain and treat diseases, as well as for providing essential sources for the herbal, pharmacy, and phytotherapy industries [11,12].

Essential oils comprise secondary metabolites that plants produce in response to external conditions. Despite being found in multiple ecological niches, their diversity, abundance, and nature vary even within the same species [13,14]. Over the last 50 years, research has provided substantial knowledge about the chemical diversity of volatile organic compounds (VOCs) in plants and their potential use as antimicrobial agents [15].

Oregano (*Origanum vulgare* L.) is known for its high concentration of thymol and carvacrol essential oils. These oils have potent antimicrobial properties and are often used with other antibiotics to treat infections and develop medicines [16,17,18]. Oregano essential oil (OEO), derived from the leaves of the *Origanum vulgare* plant, is known for its potent antimicrobial properties and diverse therapeutic benefits. OEO is utilized in natural remedies for respiratory conditions, for inflammation, and even as a general immune system booster. Rich in carvacrol and thymol, two compounds with significant antibacterial and antifungal effects, OEO has shown promise in inhibiting bacterial growth and reducing biofilm formation [16,17,18]. Essential oils (EOs) contain volatile compounds with antimicrobial properties which can effectively combat multidrug-resistant pathogens [19,20].

Various studies have shown that infusions and the essential oil of *O. vulgare* have excellent antimicrobial properties against multidrug-resistant bacterial and fungal microorganisms such as *E. coli*, *S. aureus*, *Candida albicans*, and *Pseudomonas aeruginosa* [21,22]. These studies observed that the main mechanisms of action against microorganisms include enzymatic inhibition, efflux pump inhibition, ATP depletion, inhibition of biofilm formation, and damage to the cytoplasmic membrane [21]. OEO has been discovered to have biocidal activity against up to eleven different microorganisms, including some of the most common and harmful bacterial and fungal strains, including *S. aureus*, *E. coli*, *Bacillus subtilis*, *S. epidermidis*, and *S. cerevisiae*. This is due to its main compounds: terpinene 4-ol and trans-sabinene hydrate [23,24,25]. Therefore, more knowledge about the appropriate dosage and application conditions is necessary.

This study aimed to assess the chemical composition of the essential oil extracted from the Peruvian *O. vulgare* variety and its effectiveness in controlling the growth of *S. aureus* and *E. coli* under laboratory conditions. To achieve this, the essential oil of *O. vulgare* was analyzed using GC-MS. Furthermore, the susceptibility of each strain to the essential oil was assessed, along with the minimum inhibitory concentration.

## 2. Results

### 2.1. Main OEO Compounds

The essential oil of oregano (OEO) was extracted from *O. vulgare* plants collected from the Chachapoyas central market and cultivated at 2600 masl (Taquia village). The OEO had a density of 957 mg/mL. A total of 26 compounds were identified in the oregano essential oil, with a minimum abundance of 0.1% (Table 1). Additionally, a category of other minor compounds, which were not specifically identified, collectively made up 0.65% of the oil. The essential oil of *O. vulgare* is primarily composed of monoterpenes, with the menthane monoterpenoid, 2-menthen-1-ol, being the most abundant at 36.33%. Other prominent monoterpenes included linalyl acetate (9.26%), terpinen-4-ol (9.01%), 4-thujanol (6.33%), menthene (5.81%), sabinene (5.18%), and carvacrol methyl ether (5.14%). Additionally, minor monoterpenes such as o-cymene (3.37%), beta-myrcene (1.76%), terpinolene (1.71%), D-limonene (1.36%), and alpha-thujene (1.01%) were also present. Alcoholic monoterpenes like L-alpha-terpineol (2.67%), linalool (2.27%), and thymol (1.93%) were identified. Furthermore, alpha-phellandrene (0.36%), beta-phellandrene (1.44%), and delta cadinene (0.14%) were detected, which are known to have immunomodulatory and synergistic antimicrobial effects. No diterpenes or sesquiterpenes were found in the sample. Consequently, OEO is a significant source of monoterpenes and terpenoids, contributing to its antimicrobial properties. It is worth noting that certain compounds, such as pulegone (0.56%), have reported toxicity and effects other than antimicrobial activity.

### 2.2. Bacterial Susceptibility to OEO

The preliminary antibacterial activity of OEO was investigated using the disc diffusion method (Kirby–Bauer). The inhibition zones were found to be significantly smaller compared to the reference antibiotic for both bacteria (Figure S2). Table 2 shows the mean inhibition zone diameters for each strain according to the Kirby–Bauer test. Chi-square results revealed a significant association between the antimicrobial effect of OEO and bacterial growth. The highest inhibition zone diameter (20 mm) was observed for the *S. aureus* isolate and *E. coli* ATCC 25922 when using concentrated OEO (100%), while the lowest diameter (16 mm) was for *S. aureus* ATCC 25923. The inhibition zone diameters were very similar for both strains across all OEO dilutions. Nonetheless, *S. aureus* ATCC 25923 showed less susceptibility to the 60% OEO dilution.

### 2.3. Minimum Inhibitory Concentration (MIC)

**Figure 1:**
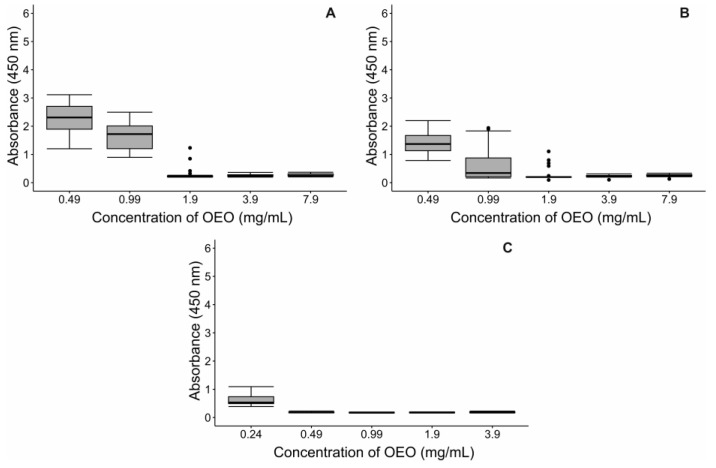
(**A**) A minimum inhibitory concentration (MIC) assay was used to assess the effect of OEO on bacterial strains. The bacterial cultures were left to incubate at 37 °C for 18 h without shaking. The absorbance values of the cell proliferation reagent WST-1 (OD: 450 nm) were represented in boxplots according to the concentrations of the OEO for (A) *S. aureus* ATCC 25923, (**B**) *S. aureus* isolated from a patient, and (**C**) *E. coli* ATCC 25922. A descriptive analysis was performed using boxplot graphs to understand the relationship between OEO concentration and optical density. In each box, the horizontal lines indicate median values, while the vertical lines (whiskers) extend to the minimum and maximum values. Dots represent outliers. The data were collected from three independent experiments.

**Figure 2:**
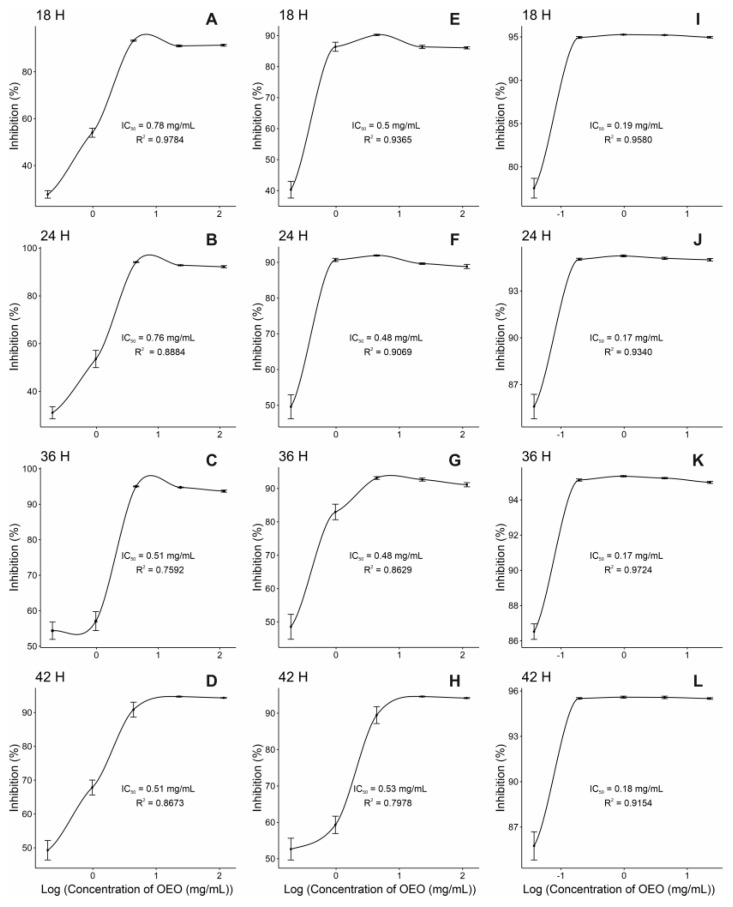
A log-logistic regression with three parameters was performed to calculate the MIC of each bacterial strain using a dose–response model (DRM). This model was used since the sigmoidal curve better fits the experimental data. The logistic regression graph denotes the IC50 and hours of exposure for *O. vulgare* essential oil on (**A**–**D**) *S. aureus* ATCC 25923, (**E**–**H**) *S. aureus* isolated from the patient, and (**I**–**L**) *E. coli* ATCC 25922. The graphs were generated with the data of the three independent experiments of cell proliferation with WST-1.

Certain limitations, such as high hydrophobicity and poor solubility, restrict the use of essential oils (EOs). To conduct the MIC assay in an aqueous environment, an emulsion containing OEO, bacterial culture medium, and Tween 20 as an emulsifier was prepared. The 1:1 ratio emulsion, which was evaluated for up to 48 h, demonstrated the best stability. Microscopic observation revealed uniformly distributed droplets of OEO with a diameter of 16.46 ± 3.70 μm (Figure S1). For MIC assays, ten-fold dilutions were performed from 100% OEO, as all concentrations resulted in inhibition halos in the Kirby–Bauer test. The MIC for the *S. aureus* isolate and the ATCC strain was 1.9 mg/mL, while for *E. coli* it was 0.49 mg/mL (Figure 1). As expected, the post-exposure effect of OEO was observed over time (Figure 2 and Table 3). However, some outliers were observed due to the highly concentrated nature of the emulsions. The IC50 for the *S. aureus* isolate was 0.51 mg/mL and 0.77 mg/mL for the ATCC strain after 18 h of exposure. At the end of the 42 h experiment, the IC50 remained around 0.5 mg/mL for *S. aureus*. For *E. coli*, the IC50 was between 0.18 and 0.19 mg/mL after 18 and 42 h of exposure, respectively (Figure 2 and Table 3). These results confirm the strong antimicrobial activity of OEO against Gram-negative bacteria, such as *E. coli*, with a lower IC50.

According to Table 4, the Kruskal–Wallis test is significant when analyzing OEO exposure time, indicating that the percentage of inhibition varies based on exposure time. The Nemenyi post hoc test reveals differences between time points. At 18 h, the OEO inhibited 89.5% of bacterial growth, and this inhibition increased to 94.2% after 42 h (Table 4). The analysis also shows a significant difference in the growth inhibition percentage between Gram-negative *E. coli* and Gram-positive *S. aureus*, with 95.1% and 91.8% inhibition, respectively (Table 4).

### 2.4. Minimum Bactericidal Concentration

**Figure 3:**
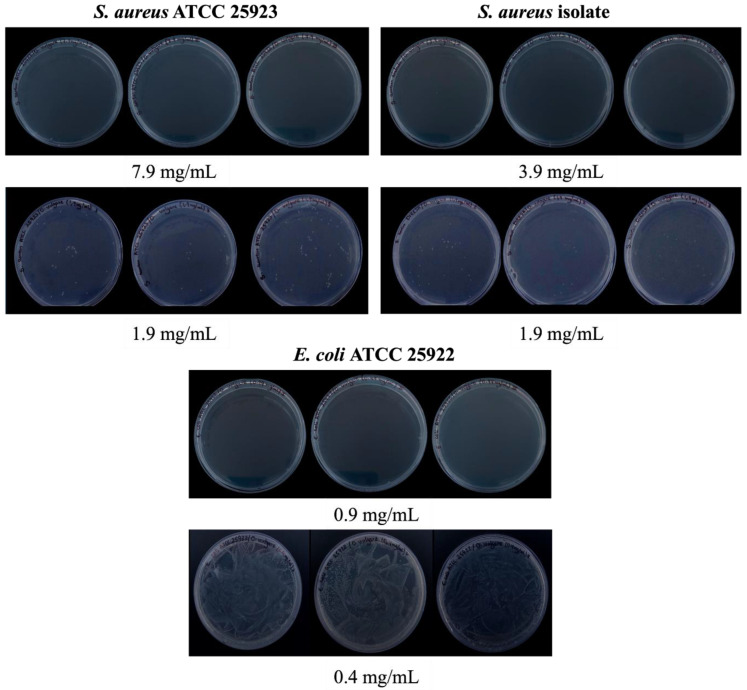
The minimum bactericidal concentration (MBC) was determined by using bacterial cultures from wells that showed no color variation in the microplate used in the MIC assay. The samples were then spread radially on Müller–Hinton agar plates using a Drigalsky loop. The plates were incubated at 37 °C for 24 h, and the concentration at which no colonies grew was considered the MBC. The experiment was conducted in triplicate to ensure accuracy.

The minimum bactericidal concentration (MBC) of OEO was tested against two bacterial strains—*S. aureus* ATCC 25923 and *E. coli* ATCC 25922—and a *S. aureus* isolate. The results revealed that for *S. aureus* ATCC 25923, the MBC of OEO was 7.9 mg/mL, while for the isolate it was 3.9 mg/mL. On the other hand, the MBC for *E. coli* ATCC 25922 was 0.99 mg/mL, which was close to the MIC result. These findings suggest that OEO has a higher bactericidal activity against Gram-negative E. coli than Gram-positive *S. aureus* with a low concentration of OEO (Figure 3).

## 3. Discussion

The chemical composition of essential oils can vary due to several factors, such as geographical origin, environmental conditions, harvest time, agricultural practices, quality of raw materials, and processing parameters [25]. In the composition of the OEO from Chachapoyas (2300 m above sea level), this study found as primary compounds 2-menthen-1-ol, followed by linalyl acetate, terpinene-4-ol, 4-thujanol, menthene, sabinene, and carvacrol methyl ether.

This study found 2-Menthen-1-ol as the most abundant compound, unlike other studies where thymol and carvacrol were among the most abundant compounds in other plant species cultured over 2945 masl [25] or compared with other Laminaceas like *Thymus vulgaris* [26]. In this case, altitude and terrain could determine the composition of the secondary metabolites. Other studies have shown that carvacrol interferes with the gene expression and adhesion factors of *E. coli* [27]. Thymol (2-isopropyl-5-methyl-phenol) has antifungal and antibacterial functions as well as multiple antioxidant, microbiological, and food preservative properties, in addition to potential applications in perfumery and cosmetics [28,29,30].

While carvacrol has not been found in oregano essential oil, a derivative called carvacrol methyl ether was identified. This compound, along with thymol methyl ether, was reported to have lower antimicrobial activity due to the lack of the hydroxyl group present in the phenolic terpenoids carvacrol and thymol [31]. However, carvacrol methyl ether was identified as a potent antioxidant [32]. The most abundant compounds discovered in this study belong to the class of oxygenated monoterpenes. These compounds are believed to have toxic effects on the function and structure of bacterial cell membranes, indicating that molecules like carvacrol have the potential to inhibit bacterial growth. Such activity has been demonstrated in active packaging materials, ensuring food safety and extending shelf life [32,33].

Several studies have demonstrated the antimicrobial activity of monoterpenes, such as terpinene-4-ol and 4-thujanol, either alone or in combination with other terpenes. These studies have shown their effectiveness against bacteria such as *Shigella flexneri* or *Pseudomonas* sp. [34,35]. It is important to note that compounds with low abundance should not be disregarded, as they can have a synergistic effect [36]. This aspect should be considered when comparing in vitro studies using isolated compounds and essential oils, which are complex products.

According to the Kirby–Bauer test, the diameter corresponding to the inhibition zone increased with a higher percentage of the essential oil. *E. coli* ATCC 25922 was the most sensitive strain, indicating that OEO could substantially affect some Gram-negative bacteria. Likewise, some studies have shown a significant inhibitory effect on Gram-positive bacteria, including *Bacillus subtilis*, *Staphylococcus aureus*, and *Staphylococcus saprophytic* [37,38]. Gram-negative bacteria have a thin layer of peptidoglycan surrounded by an external layer composed of lipopolysaccharides, which gives them additional protection and contributes to their pathogenic properties. Nevertheless, OEO could disrupt cell wall structures and, potentially, inhibit enzymes that enhance the bacteria’s pathogenicity. This is evidenced by the substantial inhibition zone, whose diameter ranged from 10 to 20 mm in the assay.

The semiquantitative MIC assay using the Wst-1 reagent is a reliable method to determine bacterial proliferation. The MIC assay of the OEO indicates a high potential for inhibiting bacterial growth against *S. aureus* (MIC: 1.9 mg/mL, MBC: 3.9 for *S. aureus* isolate) and *E. coli* (MIC: 0.49 mg/mL, MBC: 0.99 mg/mL). This finding is consistent with other studies that have reported the antimicrobial properties of other OEOs against *S. aureus* [23,25]. Other results showed that OEO exhibited antibacterial activity against *E. coli* O157:H7 with a MIC of 0.156 μL/mL [39]. It has been proposed that OEO exerts bactericidal effects by damaging cell structures, permeabilizing cellular membranes, inducing membrane potential depolarization, and oxidative stress [39].

Therefore, more studies to understand the antibacterial effect of OEO are necessary. Contrary to the findings of Smirgiotis et al., carried out with *O. vulgare* grown at 2945 masl in the Andes of Chile [25], this study found that the essential oil of *O. vulgare* grown in the Peruvian Andes had a more significant effect on *E. coli* (Gram-negative) than *S. aureus* (Gram-positive). It is important to note that the effect of OEO on specific types of bacteria in this study cannot be generalized due to the limited number of strains evaluated. Further studies involving a greater variety of bacterial species of both types are necessary. One possible explanation for this difference is the variation in the composition of both Andean OEOs. The Chilean OEO contains thymol (15.9%), z-sabinene hydrate (13.4%), and gamma-terpinene (10.6%) as the most abundant monoterpenes, while this OEO contains 2-menthen-1-ol (36.3%), linalyl acetate (9.26%), and 4-terpineol (9.01%) as the most abundant. Meanwhile, for the Peruvian OEO, differences in the abundance percentage and isomerism were found. Moreover, differences in the isomerism and the abundance of monoterpenes support the idea of differences in the mechanisms of action [40].

One potential application for OEO includes the microbial decontamination of food-contact surfaces. However, its efficacy must be evaluated since this depends on several parameters, such as target microorganisms, food-contact material, temperature, time of contact, and relative humidity [41].

Finally, it is important to conduct further research on the potential impact of OEO on other harmful microorganisms. Currently, there are gaps in the knowledge on how OEO compounds can affect enzymatic transporters that facilitate the uptake of essential nutrients, bacterial signaling pathways, and the potential development of resistance in microorganisms with long-term use of OEO. Additionally, combining essential oils from different plants may improve their effectiveness in inhibiting both Gram-positive and Gram-negative bacteria, which can help treat infectious diseases.

## 4. Materials and Methods

### 4.1. Plant Specimen

A complete fresh specimen of *O. vulgare* was collected from the Chachapoyas central market. It is well known that the site of the culture of the plant is located in Taquia village (2600 masl) with coordinates of 6°15′15″ S (latitude) and 77°50′12″ W (longitude), 30 min from Chachapoyas city. The taxonomic identification was performed in the “Kuelap” Herbarium of the Universidad Nacional Toribio Rodríguez de Mendoza de Amazonas. The specimen of *O. vulgare* from Taquia village was deposited in the herbarium with the code Kuelap–4287. The samples were dried at room temperature (13 °C to 23 °C) in the city of Chachapoyas for approximately 5 to 7 days, and when the leaves began to detach, they were stored in a dry plastic bag.

### 4.2. Essential Oil Extraction from Oregano

The dehydrated plant material was taken to the Laboratory of Engineering from the Faculty of Engineering and Agricultural Sciences—UNTRM, where the OEO was obtained through the steam distillation technique following the procedure described by Castro-Alayo et al. [41]. The OEO obtained was stored at 4 °C, protected from light, in sterile amber vials of 20 mL.

### 4.3. Chemical Composition by Gas Chromatography

The chemical composition of the essential oil of *O. vulgare* was identified following Adams, R [42] with some variations, using a gas chromatograph, model 7890B GC system, coupled to a mass detector, model 5977B MSD (Agilent Technologies^®^). The compounds were separated on a DB–5MS UI capillary column (60 m × 0.25 mm × 1.0 μm, Agilent^®^). The injector temperature was 220 °C, and 0.5 µL of the diluted essential oil (5 µL of the oil + 995 µL of hexane) was injected in splitless mode. The oven temperature program varied from 60 to 246 °C at 3 °C/min, held for 8 min, then increased to 300 °C at 5 °C/min and was held for 4.2 min. Helium was used as a carrier gas with a flow of 1 mL/min. The transfer line, ionization source, and quadrupole detector temperatures were 240, 280, and 150 °C, respectively. Masses from 40 to 600 m/z were scanned. The mass spectra obtained were compared to the NIST 2017 library using a match factor higher than 70%, and the linear retention index was calculated by injecting an alkane series (C8–C20). The calculated RI showed a maximum numerical difference of ±28 units with the RI of the literature.

### 4.4. Antimicrobial Activity

Bacterial strains: *S. aureus* ATCC 25923, *E. coli* ATCC 25922, and *S. aureus* isolated from a patient were provided by the Regional Health Directorate (DIRESA)—Amazonas. For bacterial activation, each bacterial strain was inoculated in brain heart infusion broth (BHI) and incubated at 37 °C for 16 h [43]. To evaluate antimicrobial activity, strains were inoculated on nutrient agar using the depletion method and incubated at 37 °C for 24 h. At the end of the incubation, isolated colonies were selected, suspended in brain heart infusion broth (BHI), and incubated at 37 °C for 24 h. Afterward, the turbidity of the bacterial populations was adjusted by spectrophotometry (OD600) to the scale of the McFarland nephelometer (tube No. 0.5), with a population density of 1.5 × 10^8^ CFU/mL, using a spectrophotometer UV-VIS, brand Thermo Scientific, model Genesys 10S UV-Vis.

### 4.5. Susceptibility of Each Strain Against OEO

The antimicrobial activity of OEO was evaluated by the diffusion method in Kirby–Bauer agar [44], for which the mass inoculation of bacterial suspensions was carried out (1.5 × 10^8^ CFU/mL) in Müller–Hinton plates; then, sterile 6 mm diameter discs of Whatman No. 01 filter paper impregnated with essential oil at 957, 765.6, 574.2, 382.8, and 191.4 mg/mL were placed and incubated at 37 °C for 24 h. As susceptibility controls, antibiotics were used; gentamicin (10 μg) was used for *S. aureus* and amikacin (30 μg) for *E. coli*. After the incubation, the inhibition halos (mm) were measured with a vernier. The assay was performed in triplicate.

### 4.6. Determination of Minimum Inhibitory Concentration (MIC)

Initially, stock solutions were prepared; after that, oregano essential oil (957 mg/mL), culture medium (BHI), and Tween 20 (1:0.5; 1:1; 1:2 and 1:3 for OEO) were mixed. After 48 h, the thermodynamic stability of these solutions was observed in an inverted fluorescence microscope (IX83, Olympus, Tokyo, Japan) with a magnification of 500X and high-resolution polarized light and equipped with a digital camera (Nikon D810, Tokyo, Japan), the optimal solution was in a 1:1 proportion, whose concentration of oregano essential oil was 319 mg/mL, as shown in Figure 1.

The MIC of OEO against the studied microorganisms was performed using the protocol of Pizzo et al. [45], with some variations. First, in a 96-well microdilution plate, 100 μL of OEO dilutions and 10 μL of the standardized bacterial inoculum (1.5 × 10^8^ CFU/mL) were added to be incubated at 37 °C for 18 h without shaking. Therefore, a positive control (culture medium with bacterial inoculum) and a negative control (culture medium with bacterial inoculum plus ampicillin/oxacillin solution at 5 mg/mL) were used for *E. coli* and *S. aureus*, respectively. The WST-1 reagent (Abcam, ab155902) was used as a colorimetric inhibition indicator to measure cell proliferation. The tetrazolium salts contained in the reagent are cleaved to formazan by cellular enzymes. The wavelength for measuring the absorbance of the formazan product is between 420 and 480 nm (max. absorption at about 440 nm) according to the filters available for the ELISA reader. A total of 10 μL of cell proliferation reagent WST-1 (Abcam, ab155902) was added half an hour before the interpretation, then set up at 350 revolutions per minute (RPM) for 2 min in a shaker (Dlab, SK-R330-Pro) and in the microplate reader (Biobase, EL10A) at OD450. Furthermore, the bacterial growth was monitored at 24, 36, and 42 h. The assay was performed in triplicate.

### 4.7. Determination of the Minimum Bactericidal Concentration (MBC)

The minimum bactericidal concentration (MBC) was determined using Paiano’s protocol [46]. Ten microliters were taken from the ones showing no color variation. The samples were then spread radially on Müller–Hinton agar plates using a Drigalsky loop. The plates were incubated at 37 °C for 24 h, and the concentration at which no colonies grew was considered as the MBC. The experiment was conducted in triplicate to ensure accuracy.

### 4.8. Data Analysis

Before analyzing the data, a Kolmogorov–Smirnov normality test and Bartlett’s homogeneity test were carried out to determine the appropriate statistical tests needed [44]. A Kruskal–Wallis test was run to evaluate significant differences in the inhibition halo (susceptibility) of each established OEO dilution for each strain in the Kirby–Bauer test. Nemenyi’s nonparametric post hoc test was used since the data were balanced [47]. Several analyses were performed to determine and evaluate the MIC. First, a descriptive analysis was performed using boxplot graphs to observe the general behavior of the established OEO concentrations versus the optical density (OD) obtained at 450 nm. Subsequently, the percentage inhibition was calculated from the optical densities found in the experiments using the following equation [48]:

Inhibition (%) = (OD450 control − OD450 sample)/OD450 control × 100.

where OD450 = optical density 450 nm.

Then, the MIC of each bacterial strain was calculated using a dose–response model (DRM) based on a log-logistic regression with three parameters. This model was used since the sigmoidal curve better fits the experimental data [49,50]. Finally, the percentage inhibitions of exposure times and strains were compared to check if there were significant differences. For this purpose, the Kruskal–Wallis test was applied for the two factors above. When the Kruskal-Wallis test indicated significant differences, the Nemenyi nonparametric post hoc test was used to perform multiple comparisons and determine the differences between levels of each factor, as the data were balanced. [47]. All statistical analyses were performed at a significance level of *p* <0.05 using R software version 4.3.1 [51].

## 5. Conclusions

The essential oil of oregano from the highlands of Peru contains compounds with known antimicrobial properties, such as 2-menthen-1-ol, linalyl acetate, terpinene-4-ol, 4-thujanol, menthene, sabinene, and carvacrol methyl ether. This suggests that oregano essential oil (OEO), combined with other compounds, could be a novel strategy to combat bacteria, including antibiotic-resistant strains. The environment and altitude where oregano is grown can influence the composition of its antimicrobial molecules. It is important to compare these findings with those for other oregano oils from different origins and to test the effects on antibiotic-resistant microorganisms. This research could have positive implications for animal and human health as well as public health. Further studies are needed to determine the effects of essential oils on other microorganisms of public health concern.

## Figures and Tables

**Table 1:** Chemical composition of *O. vulgare* essential oil by GC-MS.

Retention Time	Compound	Abundance (%)	RI Cal ^a^	RI Lit ^b^	Fragments *m/z*
31.06	2-Menthen-1-ol	36.33	1117	1126	43.0; 71.0; 81.0
37.89	Linalyl acetate	9.26	1250	1257	93.0; 43.0; 121.0
35.33	Terpinen-4-ol	9.01	1198	1177	71.1; 93.2; 111.2
29.24	4-Thujanol	6.33	1083	1075	93.2; 71.1; 43.1
26.26	Menthene	5.81	1028	1017	121.0; 93.0; 136.0
23.85	Sabinene	5.18	984	974	93.2; 91.2; 77.1
37.73	Carvacrol methyl ether	5.14	1247	1244	149.0; 164.0; 91.0
26.66	o-Cymene	3.37	1036	1022	119.0; 134.0; 117.0
35.92	L-.alpha.-Terpineol	2.67	1210	1190	59.0; 93.0; 121.0
37.19	Thymol methyl ether	2.36	1236	1235	149.0; 164.0; 91.0
30.32	Linalool	2.27	1103	1099	93,2; 71,1; 41,1
40.53	Thymol	1.93	1304	1291	135.0; 150.0; 91.0
24.21	.beta.-Myrcene	1.76	991	991	93.0; 69.0; 41.0
30.03	Terpinolene	1.71	1097	1088	121.2; 93.2; 136.2
27.21	.beta.-Phellandrene	1.44	1046	1031	93,0; 91,0; 77,0
26.97	D-Limonene	1.36	1041	1018	68.0; 93.0; 67.0
21.11	alpha-Thujene	1.01	934	929	93.0; 91.0; 77.0
	Other compounds	0.65	-	-	-
38.26	Pulegone	0.56	1258	1237	81.1; 152.2; 67.2
27.35	Z-beta-Ocimene	0.44	1048	1038	93.0; 91.0; 79.0
25.71	.alpha.-Phellandrene	0.36	1018	1005	93.0; 91.0; 77.0
36.1	trans-Piperitol	0.27	1214	1208	84.2; 93.2; 91.2
35.09	endo-Borneol	0.25	1194	1167	95.2; 110.2; 41.1
23.52	1-Octen-3-ol	0.15	978	980	57.0, 43.0; 72.0
33.99	(-)-Menthone	0.14	1173	1154	112.0; 69.0; 139.0
51.21	delta-Cadinene	0.14	1542	1524	161.0; 119.0; 134.0
34.64	Levomenthol	0.1	1185	1175	71.1; 95.2; 81.1

a: calculated retention index, b: literature retention index.

**Table 2:** Kirby–Bauer assay. Measurements of inhibition zones (mm) of *O. vulgare* essential oil against *S. aureus* ATCC 25923, *S. aureus* isolate, and *E. coli* ATCC 25922.

Density Dilution (mg/mL) [%]	*S. aureus* ATCC 25923	*S. aureus* Isolate	*E. coli* ATCC 25922
	(χ^2^ = 16.31; *p* = 0.006)	(χ^2^ = 16.59; *p* = 0.005)	(χ^2^ = 16.18; *p* = 0.006)
957 [100]	16.7 (1.1) *	19.7 (0.6) *	20.7 (1.2) *
765.6 [80]	14.3 (1.5) *	18.3 (1.2) *	19.0 (2.0) *
574.2 [60]	11.3 (0.6) *	15.3 (0.6) *	14.7 (1.2) *
382.8 [40]	9.3 (1.2) ⍏	10.7 (0.6) *	11.0 (1.0) ⍏
191.4 [20]	7.7 (1.5) ⍏	8.3 (0.6) ⍏	10 (0.3) ⍏
Susceptibility control	26 (0.1) (a)	26 (0.1) (a)	29 (1.5) (b)

The values represent the mean and (SD) of three independent assays. Susceptibility controls: (a) 10 ug gentamicin, (b) 30 ug amikacin. * Significant with respect to susceptibility control. ⍏ Significant with respect to higher dilutions. Nemenyi post hoc test (*p* < 0.05).

**Table 3:** Inhibition percentage and IC50 of OEO against *S. aureus* ATCC 25923, *S. aureus* isolate, and *E. coli* ATCC 25922.

Microorganism	Time	% Inhibition						IC 50%	CI 95%
		7.9 mg/mL	3.9 mg/mL	1.9 mg/mL	1.0 mg/mL	0.5 mg/mL	0.2 mg/mL		
*S. aureus* ATCC	18	91.4	91.1	93.3	54.0	27.7	-	0.77	0.72–0.82
	24	92.2	92.8	94.1	53.6	31.1	-	0.76	0.69–0.84
	36	93.7	94.7	95.0	57.1	54.4	-	0.51	0.41–0.61
	42	94.4	94.7	90.9	67.8	49.3	-	0.51	0.45–0.56
*S. aureus* isolate	18	86.1	86.4	90.3	86.4	40.3	-	0.5	0.49–0.52
	24	88.8	89.6	91.9	90.7	49.6	-	0.48	0.44–0.53
	36	91.1	92.7	93.2	83.0	48.5	-	0.47	0.44–0.50
	42	94.1	94.6	89.5	59.3	52.6	-	0.53	0.43–0.63
*E. coli* ATCC	18	-	95.0	95.2	95.3	94.9	77.5	0.19	0.13–0.24
	24	-	95.0	95.1	95.2	95.0	85.6	0.17	0.07–0.27
	36	-	95.0	95.2	95.3	95.1	86.5	0.17	0.11–0.24
	42	-	95.6	95.6	95.6	95.5	85.7	0.18	0.05–0.29

IC50%: mean inhibitory concentration; CI 95%: 95% confidence interval.

**Table 4:** Kruskal–Wallis analysis determines differences in inhibition percentages among time points (hours post-exposure) and bacteria strains.

Differences Among Time Points	
Time	Median
18	89.593 ^a^
24	91.450 ^a,b^
36	92.921 ^b,c^
48	94.243 ^c^
**Differences Among Bacteria**	
Strains	Median
*S. aureus* isolated	89.334 ^a^
*S. aureus* ATCC	91.827 ^a^
*E. coli* ATCC	95.141 ^b^

The Nemenyi post hoc test was conducted subsequent to identifying significant differences with the Kruskal–Wallis test. The letters (^a,b,c^) denote the outcomes of the test. If medians share at least one letter, it indicates that there are no significant differences between them. Conversely, if the medians have different letters, it signifies that there are statistically significant differences. Nemenyi post hoc test (*p* < 0.05).

## Data Availability

The authors confirm that all the data supporting this study are available within the article.

## Supplementary Materials

The following supporting information can be downloaded at https://www.mdpi.com/article/10.3390/ph17111430/s1: Figure S1: Emulsification of the EO of *O. vulgare*: Microscopic images of the various emulsions prepared using different component ratios. BHI broth + Tween 20 vs. essential oil in the following ratios: (A) 1:0.5, (B) 1:1, (C) 1:2, and (D) 1:3. Figure S2: Disc diffusion assay evaluating the effect of different concentrations of OEO on bacterial cultures of *E. coli* and *S. aureus.* PC: positive control, gentamicin (10 μg) for *S. aureus* and amikacin (30 μg) for *E. coli*. SC: sterility control.
