# Supplementary material for: Antimicrobial Activity of Origanum vulgare Essential Oil against Staphylococcus aureus and Escherichia coli
Source: Pharmaceuticals (Basel). 2024 Oct 25;17(11):1430. doi: 10.3390/ph17111430 (PMC11597097; doi:10.3390/ph17111430)
Supplement: Supplementary file 1 [file pharmaceuticals-17-01430-s001.zip › pharmaceuticals-3148887-supplementary.pdf]

Supplementary Materials

Antimicrobial activity of *Origanum vulgare* essential oil against *Staphylococcus aureus* and *Escherichia coli*

Sonia Tejada - Muñoz[1,2,*], Denny Cortez[1], Jesús Rascón[1,3], Segundo G. Chavez[3], Aline C. Caetano[3], Rosa J. Díaz Manchay[4], Julio Sandoval-Bances[1], Sonia Huyhua[1,2], Lizandro Gonzales[5], Stella M. Chenet[1,6], Rafael Tapia-Limonchi[1,6*]

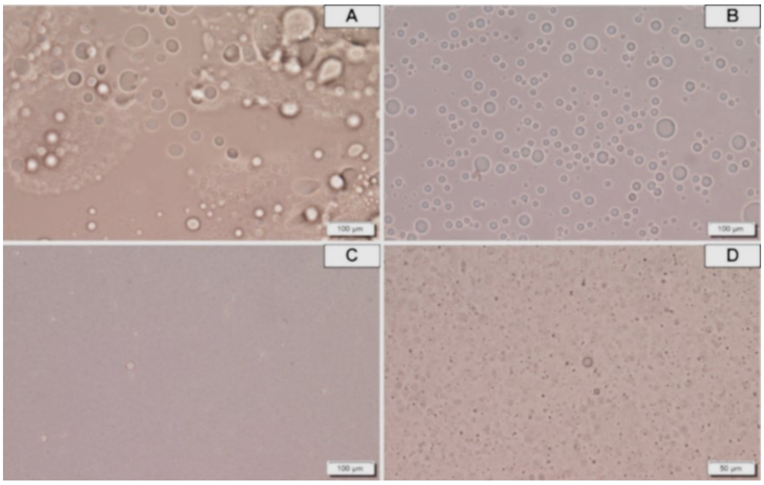

Figure S1. Emulsification of the EO of *O. vulgare*: Microscopic pictures of the different emulsions prepared at different components ratio. BHI broth +Tween20, and essential oil in a 1:0.5 (A), 1:1 (B), 1:2 (C), and 1:3 (D).

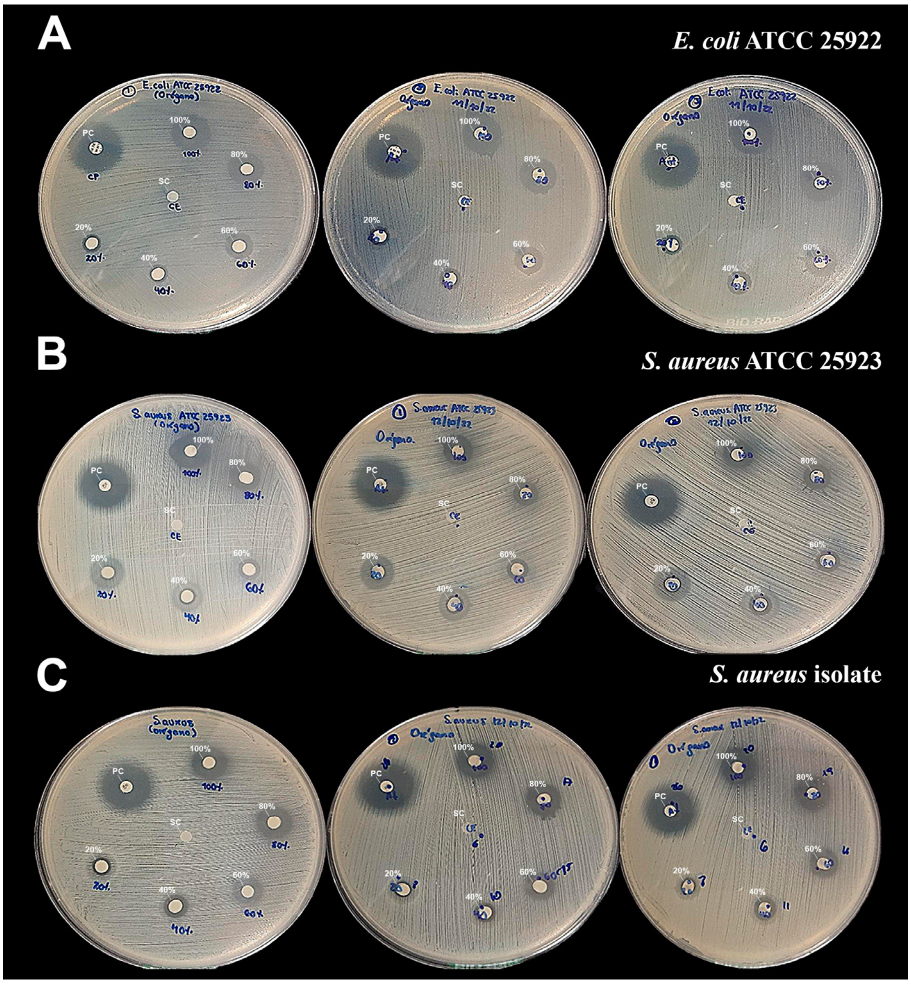

Figure S2. Disc diffusion assay evaluating the effect of different concentrations of OEO on bacterial cultures of *E.coli* and *S.aureus*. PC: positive control, Gentamicin (10 µg) for *S. aureus* and Amikacin (30 µg) for *E. coli* . SC: sterility control.
